# Ecosystem Service Valuation Assessments for Protected Area Management: A Case Study Comparing Methods Using Different Land Cover Classification and Valuation Approaches

**DOI:** 10.1371/journal.pone.0129748

**Published:** 2015-06-18

**Authors:** Charlotte E. L. Whitham, Kun Shi, Philip Riordan

**Affiliations:** 1 Wildlife Institute, School of Nature Conservation, Beijing Forestry University, Haidian District, Beijing, China; 2 Department of Zoology, University of Oxford, Oxford, United Kingdom; 3 Wildlife Without Borders, Oxfordshire, United Kingdom; The Ohio State University, UNITED STATES

## Abstract

Accurate and spatially-appropriate ecosystem service valuations are vital for decision-makers and land managers. Many approaches for estimating ecosystem service value (ESV) exist, but their appropriateness under specific conditions or logistical limitations is not uniform. The most accurate techniques are therefore not always adopted. Six different assessment approaches were used to estimate ESV for a National Nature Reserve in southwest China, across different management zones. These approaches incorporated two different land-use land cover (LULC) maps and development of three economic valuation techniques, using globally or locally-derived data. The differences in ESV across management zones for the six approaches were largely influenced by the classifications of forest and farmland and how they corresponded with valuation coefficients. With realistic limits on access to time, data, skills and resources, and using acquired estimates from globally-relevant sources, the Buffer zone was estimated as the most valuable (2.494 million ± 1.371 million CNY yr^-1^ km^-2^) and the Non-protected zone as the least valuable (770,000 ± 4,600 CNY yr^-1^ km^-2^). However, for both LULC maps, when using the locally-based and more time and skill-intensive valuation approaches, this pattern was generally reversed. This paper provides a detailed practical example of how ESV can differ widely depending on the availability and appropriateness of LULC maps and valuation approaches used, highlighting pitfalls for the managers of protected areas.

## Introduction

Understanding ecosystem service provision and valuation across spatial and temporal scales is vital to linking environmental conservation and human well-being [1–4]. Generally, it is accepted that by understanding the ability of different land-use and land cover (LULC) components to directly and indirectly provide goods and services to people, such services provided by nature can be appropriately managed and protected (e.g. [5]). The methods for classifying LULC across space and time are therefore also important in developing our understanding of ecosystem service provision. This is highlighted by standard methods for valuing ecosystem services that derive estimates for particular services in an area, and then extrapolate to other areas of similar habitat type [6,7].

The process of deriving spatial patterns of ecosystem service value (ESV) is complicated by the plethora of classification approaches available for building LULC databases [8]. Additionally, many economic methods have been developed for valuing the ecosystem services themselves (e.g. [9]). Despite the plausibility of knowing which LULC classification or economic valuation techniques might be most accurate, some may be more appropriate under specific conditions. Some may also be more accessible than others, particularly under skill, time or financial limitations [10]. This means that the most accurate or appropriate techniques are not always adopted, and so we run the risk of decision-makers being misled [5,6,11]. It is vital therefore, for us to understand the effects of different valuation methods on the results on which management decisions are based.

Ecosystem service value is known to vary temporally [12]. However, the focus of this study considered their spatial variation over a specific time period. Here we examine approaches used to calculate the value of different management zones in and surrounding a National Nature Reserve in a biodiversity hotspot of Southwest China (Myers, 1988 in: [13]). By assessing the ecosystem services provided, important management zones were identified and prioritized for action. Conservation management intending to protect the unique biodiversity within and surrounding this Nature Reserve must also take into consideration the needs and well-being of local communities living close to its borders. It therefore provides a highly relevant case study to assess the effects of using various methodologies for measuring the benefits provided to society from nature.

Two different LULC maps and three different valuation approaches were used, resulting in a total of six possible ESV measurement approaches. These approaches incorporate a range of potential methodological differences driven by the availability of resources, the use of previously accepted standards of valuation techniques and critically, knowledge of social value at a local scale ([14] but see [15]). Using the most accurate methods was not the main criteria for selecting the six valuation approaches adopted here. Other important work has focused on improving the accuracy of different approaches and furthering state of the art techniques, which for example, are able to take temporal variation [12] or spatial heterogeneity [16] into account. In the absence of an easily accessible dataset of such accuracy, we sought to design approaches that would be available and accessible to the Nature Reserve management authority. Through collaborating with this authority for over two years, our direct observation, and from discussion with Nature Reserve management staff, we can confirm their lack of access to resources for economic valuation assessment (Xiao, JH, *pers comm*.). Therefore alternative methods for this study requiring more data-, time- and skill-intensity were not used as they were deemed not to be widely available (For example, using very high resolution remote sensing data such as that used by Yu et al., (2006) [17]). It should also be noted that the purpose of this work was not to focus on the actual estimations of ecosystem service value themselves. These would generally be recorded as potential economic tradeoffs associated with comparison of values under two different scenarios, for example [5]. Rather, our indicators of present total value were used to make comparisons between different management zones and therefore across space. Using these approaches, our paper could provide a useful and practical insight into what results can be yielded from methods and data that are available to nature reserve managers.

Our main objective was to explore the effects of different LULC classifications as well as effects of different valuation approaches (using data from both social surveys and previously acquired datasets) on ESV measurements across space. In this way we did not use the same technique at different time intervals (e.g. [18]) but were using different techniques simultaneously. More specifically, we aimed to identify the different ways by which results from these various approaches could be interpreted within a protected area management context.

## Materials and Methods

### Study Site

**Fig 1:**
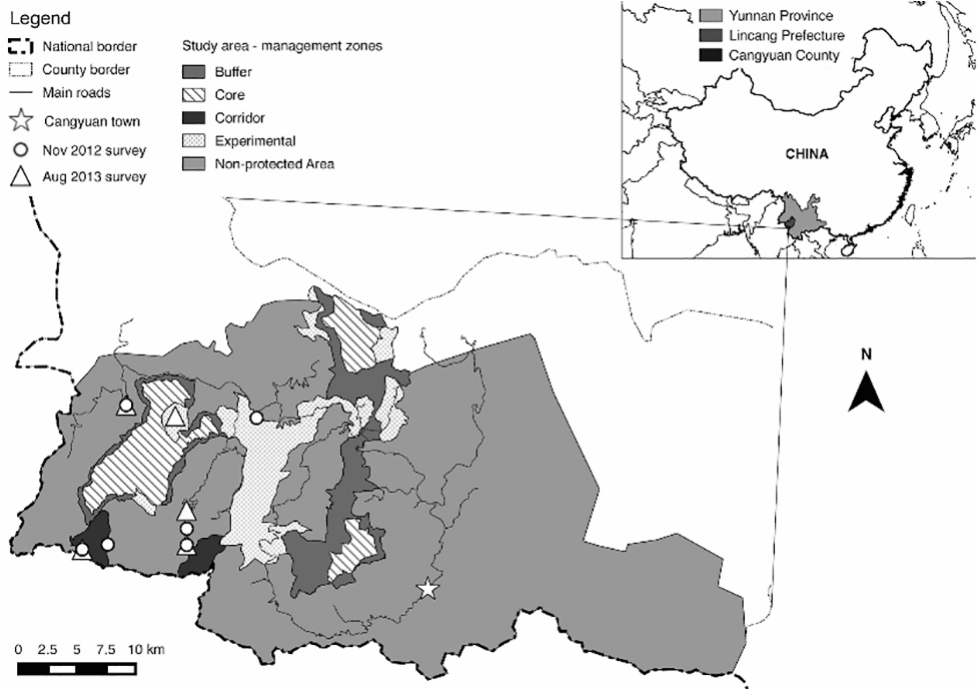
Study site location. Location of study site and fieldwork in southwestern Yunnan province with delineation of different management zones of the protected area and location of social surveys conducted in November 2012 and August 2013 (Located between 23° 4' 46.19" and 23° 23' 49.92" N & 98° 53' 1.67" and 99° 31' 30" E).

The study area (1,336.18 km^2^) covers over half of Cangyuan county in the Lincang Prefecture of China’s Yunnan Province, and borders the Republic of the Union of Myanmar to the west and south (Fig 1). The study area was delineated to incorporate Nangunhe Nature Reserve (NGH) and the extent of activity of its management authority, which also includes areas outside of the Nature Reserve. Permission to conduct the surveys mentioned below was obtained from the relevant local State Forestry Administration, the Forestry Department of Yunnan Province, and the Nangunhe Nature Reserve management authority, Cangyuan office.

The Nature Reserve itself is one of southern China’s most well-known protected areas, harbouring a wide diversity of flora and fauna within its tropical forests. This includes over 430 terrestrial vertebrate species [19,20]. For example, NGH is known to be home to several large rare mammals including the Asian elephant (*Elephas maximus*) [13,21] and several small and medium-sized felid species (e.g. Clouded Leopard (*Neofelis nebulosa*) [22] and Leopard (*Panthera pardus*)[20]). The reserve is also a strategic site for the restoration of Indo-Chinese tiger (*Panthera tigris corbetti*) populations in China [20].

NGH follows design guidelines for China’s National Nature Reserves and is divided into three management zones: Core, Buffer, and Experimental [23]. Additional “Non-protected area” and “Corridor” zones were also delineated in our study to include the entire study area. Corridor areas have not currently undergone any specific management, but have been highlighted in the NGH management plans as future potential sites for habitat restoration and ecological corridor creation between China and Myanmar [20]. Each of these five zones is managed slightly differently and different laws are applied within each of them (see [23] for more information). For example, harvesting of firewood or any non-forest product is forbidden within the Core and Buffer zones, but not in the Experimental zones. The zonation therefore provides an ideal management context in which to interpret ESV measurements.

The study area contains a population of approximately 98,700 people [24]. Cangyuan county is one of only two Autonomous counties in China dominated by the Wa ethnic group (85.1% of the county population), which attaches cultural importance to the area. Villages are dispersed throughout all areas outside of the NGH Core zone, with some small villages and farmland remaining in Buffer and Experimental zones of the Nature Reserve. A tarmacked road provides links from the main urban centre of Cangyuan town itself to most of the main villages within the study area, with some smaller sub-villages only accessible by dirt roads.

The main livelihood for people living within the study area is agriculture, primarily cassava, sugarcane, tea, rubber (*Hevea brasiliensis*) and in some parts tobacco. Rubber plantations in particular have become increasingly common, following a growing expansion of the crop across other parts of the province [25].

### Social Surveys

In November 2012, a pilot social survey was conducted in seven sub-villages within the study area (Fig 1), exploring human-wildlife interactions and in particular, ecosystem service provision at the household level. During focus group meetings in each sub-village (with normally 4–10 local villagers present, including the sub-village leader and local forest warden staff) emphasis was given to which particular ecosystem services were most important to them and their community. This information was then used for constructing a locally-relevant valuation approach, where only those services considered important to local people, as confirmed through this survey, were valued. In every sub-village the following three services were identified: 1.) Direct-use services (such as firewood, wild vegetables, bamboo and access to water); 2.) Clean air and 3.) Cultural value (this was often referred to when respondents mentioned that the study area is where they are from and where they belong). Despite limited tourism in the region, local people did not identify this as important for them.

In August 2013, a more detailed social survey was carried out in 171 households across five different sub-villages within the study area (Fig 1). These sub-villages were selected as part of a wider study and unfortunately a larger sample was beyond our time limitations. All households within each sub-village were interviewed unless a representative from a particular household was not available at the time of interview. In these cases, data from that household was not included, although this only meant three households were excluded in total. These surveys contained detailed questions regarding farmland tenure (e.g. crop type, farm area (km^2^), annual crop yield and annual income from crop sales), and harvesting of timber and non-timber forest products (including annual harvest amount (kg) and value (Chinese Yuan—CNY) of firewood, bamboo, wild vegetables and any other non-agricultural products collected in the area) (See S1 File for raw data). From these data we were able to calculate the average income per km^2^ for different crops, timber and other non-forest products harvested across the study area. These data were then used as estimations for crop production and direct-use ecosystem service value coefficients per unit area of land (See S1 Table). In selecting households for the surveys, key informants and local experts assured us that the estimates for crop production are highly unlikely to differ significantly across the study area, satisfying us that our sample was representative. We do acknowledge however, that estimates based on the harvesting of firewood and non-forest products have potentially been inflated.

### Calculating Land-use Land cover (LULC) and Ecosystem Service Value (ESV)

Two different LULC maps and three different valuation approaches were used. The framework for making ESV measurements was adapted from Troy & Wilson (2006) [6]:

- Define the study extent;
- Build LULC maps with appropriate LULC typologies for ES valuation;
- Define valuation approaches and calculate ESV per unit area coefficients to be assigned to LULC classes;
- Calculate and map ESV across LULC classes.

Using this framework, we have to assume that land within each LULC class is homogenous across space and time. We also have to assume that ecosystem service provision remains constant over time. This assumption of uniform ecosystem service value across LULC classes has been noted to inadequately represent their true heterogeneity [26]. However, obtaining such accuracy would require much higher information costs [16], not available for this project. Furthermore it was the purpose of this exercise to observe which results would be obtained using the most accessible data and techniques, and not the most accurate approaches. All map preparations and spatial analyses were conducted using Quantum GIS (QGIS) Version 2.2.0-Valmiera (2013) and Geographical Resources Analysis Support System (GRASS) Version 6.4 (2012).

### LULC Maps

The first LULC map was built using free and accessible global data from Gong et al., (2013) [27,28], offering a cheap and simple method of gaining LULC data that was originally built on a global dataset (Table 1). This 30m-resolution global land cover map (also known as Finer Resolution Observation and Monitoring of Global Land Cover, or FROM-GLC) was created using Landsat Thematic Mapper (TM) and Enhanced Thematic Mapper Plus (ETM+). To process this data into the LULC map, after downloading data for the specific area (i.e. MODIS tile 27h 6v), the raster map was then re-projected and clipped appropriately, according to the study area extent and location. A Union module was used in QGIS to combine the FROM-GLC layer with the management zone polygons, so that a final polygon layer with attributes for both LULC class and management zone was produced. This LULC map will hereafter be referred to as the “FROM-GLC” map.

**Table 1:** Brief description of the six ESV measurement approaches used (two LULC maps and three economic valuation approaches).

Valuation approach	LULC map	Economic Valuation approach
1	FROM-GLC map (derived from a 30m resolution global dataset [27,28]	Val1 –using LULC class coefficients from Costanza et al., (1997) [1]
2	FROM-GLC–as above	Val2 –As for Val1, except any coefficients for food production or raw material extraction were amended with locally acquired data. Also, local knowledge was used wherever possible to ascertain the inclusion/exclusion of services according to the local situation.
3	FROM-GLC–as above	Val3 –Only the following services were included after having been identified as most important according to responses from local people in a social survey: direct-use services, clean air, water supply and regulation and cultural value.
4	Modified-LULC map (composite created from Google Earth Imagery, forest cover data from Hansen et al., (2013) [29], and local maps provided by NGH Nature Reserve–LULC classes were chosen to be more locally appropriate)	Val1 –as above
5	Modified-LULC–as above	Val2 –as above
6	Modified-LULC–as above	Val3 –as above

The second LULC map was constructed using a much more labour-intensive but locally-relevant approach to contrast with the former FROM-GLC map (Table 1). Firstly, we wanted to identify areas of rubber plantations–one very important LULC class for this particular locality in terms of a raw material-producing service. This class was not included in the FROM-GLC map. Secondly, despite requiring time and skill, it was necessary to hand-digitize many of the key LULC classes rather than rely on computer algorithms such as those built for the FROM-GLC map [30]. We also wished to take advantage of accessibility to any other reliable data sources that were able to provide more detailed, locally-derived spatial data than what we had with the FROM-GLC layer. Details of these sources are provided below. We then used a conflation technique to combine these multiple geographic data sources to build the modified-LULC map using all available data.

Google Earth imagery (from Google Earth 7.1; Google Inc. 2013) was used at an eye altitude of 1.70–2.58 km to hand-digitize water bodies (not rivers), farmland, infrastructure, bare land, grassland, rubber plantations and obvious scrub land. These data were from 2010, the most recent data available through Google Earth. During the 2012 and 2013 field visits, location data was also collected for training areas (a total of 65 data points), which were used to ground-truth and confirm these different LULC classes. To classify the remainder of the study area, a difference geoprocessing module was used in GRASS to create a mask that included all areas not manually digitized. For the non-digitized areas, we could then extract data from a global forest layer [29]. This used imagery from 2012 and defined percentage canopy closure per output grid cell for all vegetation taller than 5m in height. This forest cover layer was then converted to a vector layer where Forest defines ≥30% canopy cover and Scrub defines < 30% canopy cover.

The hand-digitized layer was combined with the Forest/Scrub layer and with a digitized roads/river layer and management zone layer (produced in 2009) provided by NGH Nature Reserve, resulting in a final composite modified-LULC map (hereafter referred to as the modified-LULC map).

### Ecosystem Service Economic Valuation

In common with many studies (e.g. [18, 31–35]) in defining valuation approaches and calculating ESV per unit area coefficients, we employed the methodologies and calculations developed by Costanza et al., (1997) [1]. In their approach Costanza et al., (1997) [1] calculated the global value from 17 ecosystem services for 16 biomes. To calibrate the data here with their coefficients, each of our LULC classes were matched with an appropriate biome, and value coefficients were standardized to 2013 CNY equivalents per unit area (Details provided in S2 Table and S3 Table). Although these estimates are likely to have changed since they were first published in 1997, they still represent a dataset most easily accessible to us at the time of analysis.

Of the three valuation approaches used here, only one, Val1, simply used the exact high and low estimates provided by Costanza et al., (1997) [1]. Val2 was designed to make these estimates more relevant to the local situation, using the same structure as that of Costanza et al., (1997) [1], but substituted all “Food production” values with calculations from our social survey data (S1 Table). Other categories were also amended using expert, local knowledge of the area to judge the relevance of inclusion/exclusion of some services (e.g. where it was known that no recreation services are realised, the recreation value included by Costanza et al., (1997) [1] for that biome, was removed from that particular respective LULC class). Some services, such as ‘gas regulation’ for example, could not be re-calculated to produce more locally relevant estimates as the necessary data for this was not collected or available. The Val2 approach therefore provided an assessment of all ecosystem services (of local, regional and global relevance), but with as many coefficients as possible calculated with locally relevant data.

Val3 attempted to amend the estimates from Costanza et al., (1997) [1] even further, only using estimates for those services identified as locally important from social surveys. These included direct-use services (estimated using social survey data), clean air (using gas regulation value estimates from Costanza et al., (1997) [1] and water supply, water regulation and cultural value estimates (also estimated from Costanza et al., (1997) [1]).

The three valuation approaches were combined with the typologies of the two different LULC maps. To demonstrate how this was done and how the six approaches differ, we provide the following example:

The FROM-GLC map has a land class termed Broadleaf forest. We have sub-divided this class into broadleaf forest inside the Core and Buffer zones, and broadleaf forest in all other management zones (including non-protected area). Firstly, this is because we know from the NGH management authority that extraction of goods (e.g. firewood) is not allowed within the Core and Buffer zones [23], so it allows us to include or exclude the valuation of such goods, as appropriate. Secondly, the Costanza et al., (1997) [1] methods did not use Broadleaf forest as a specific class, but rather had two biomes that we could use as surrogates in this example: General forest or Tropical forest. Local expert knowledge tells us that tropical forest is more likely to be found within the Core and Buffer zones, whereas identified broadleaf forest outside of those zones is more likely to be a combination of tropical and temperate forest types. Therefore, we used estimates from the Tropical forest category [1] for Broadleaf forest inside the Core and Buffer zones and estimates from the General forest category [1] for Broadleaf forest outside of the Core and Buffer zones.

We valued the Broadleaf forest LULC class inside the Core and Buffer zones as follows:

- For Val1 we used the exact high and low estimates from Tropical forest as reported by Costanza et al., (1997) [1].
- For the Val2 approach, with the knowledge that no recreation, or raw product and food extraction occurs within this part of the protected area [20, 23], we used the high and low estimates from Tropical forest minus estimates for Recreation, Food production and Raw products [1].
- Finally, for the Val3 approach, we summed together estimates from the Tropical forest category [1] for Water supply, Water regulation, Culture and Gas regulation.

As for the LULC classifications, with these three economic valuation approaches our aim was not to find the most accurate method that most closely reflected the truth. Rather, we took care to employ the most accessible methodologies, amending them as appropriately as possible for the local situation.

Following a similar approach to Troy and Wilson (2006) [6], these value coefficients were multiplied by the areas of each LULC class within each management zone to get the total value *(V)* of that LULC class *(i)* within a management zone *(a)* (*V (ES*_*ia*_ *)*):

$$V(ES_{ia})=\sum_{k=1}^{n}A(LU_{ia})\times V(ES_{kia})$$

Where A (LU_ia_) = area of LULC class (i) in management zone (a); and V (ES_kia_) = annual value per km^2^ for ecosystem service type (k) generated by LULC class (i) within management zone (a).

## Results and Discussion

### Different Base Layers for Valuation–Comparing LULC Maps

**Fig 2:**
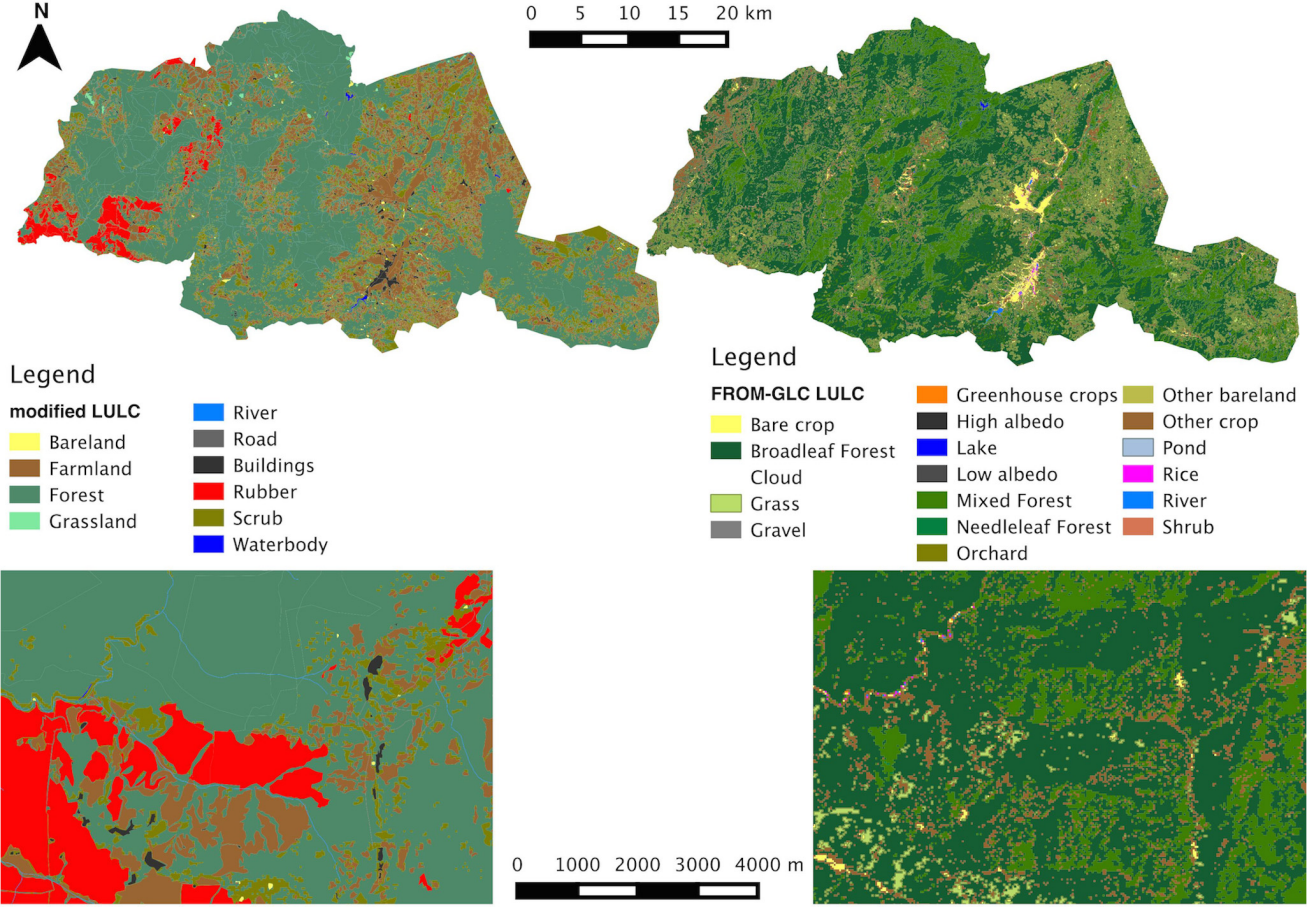
Land use land cover maps. FROM-GLC and modified-LULC maps using the extent of the study area and including a zoomed image at a common location.

The LULC maps function as the base layers from which ESV assessments can be made. The presence of rubber plantations and buildings in the modified-LULC map and its absence in the FROM-GLC map is a clear difference between the two classifications (Fig 2), although there were also more subtle differences due to the two LULC class typologies used (Table 2).

**Table 2:** Area (km^2^) and proportion of study area (%) taken up by different classes for each LULC map.

FROM-GLC Land classes	Area (km^2^)	Proportion of study area (%)	Modified-LULC Land classes	Area (km^2^)	Proportion of study area (%)
**Pond, Lake & River**	1.373	0.102	**Waterbodies & River**	4.892	0.366
**Gravel, High & Low albedo**	0.279	0.021	**Road & Buildings**	16.441	1.231
**Orchard, Greenhouse crops, Rice & Other crop**	156.670	11.725	**Farmland**	251.957	18.856
**Bare crop & Other bare land**	31.585	2.363	**Bareland**	1.527	0.114
**Shrub**	0.210	0.016	**Scrub**	207.678	15.542
**Cloud**	0.221	0.017	**Rubber**	36.531	2.734
**Grassland**	56.719	4.245	**Grassland**	1.487	0.111
**Mixed, Needleleaf & Broadleaf Forest**	1089.123	81.51	**Forest**	815.705	61.046

LULC class typology differed between the two LULC maps and so not all categories are comparable (e.g. Rubber class is present for the modified-LULC map but absent from the FROM-GLC map).

Most of the study area is made up of Forest, although the extent is greater in the FROM-GLC compared to the modified-LULC map (81.51% and 61.05% of the study area respectively). Other significant differences include the greater presence of Grassland in the FROM-GLC than in the modified-LULC map (4.25% and 0.11% respectively). In the modified-LULC map, areas of Rubber, Farmland, Water bodies & Rivers, Buildings and Scrub are much greater than in the FROM-GLC map. These differences are also present when looking at the area of comparable LULC classes across different management zones (S4 Table and S5 Table). These patterns might be expected when we consider the different LULC typologies used: For example, the FROM-GLC map lacks a Rubber class which means this land cover type could be lost within its large proportion of defined Forest class. Therefore the FROM-GLC map identifies a greater area classified as Forest in comparison to the modified-LULC map. The map resolutions may also have had an influence. For example, many roads, rivers and buildings have not been detected using the clustering and classification techniques used at a 30m resolution in the FROM-GLC map, but could be identified in the modified-LULC map.

### ESV Across the Entire Study Area

**Fig 3:**
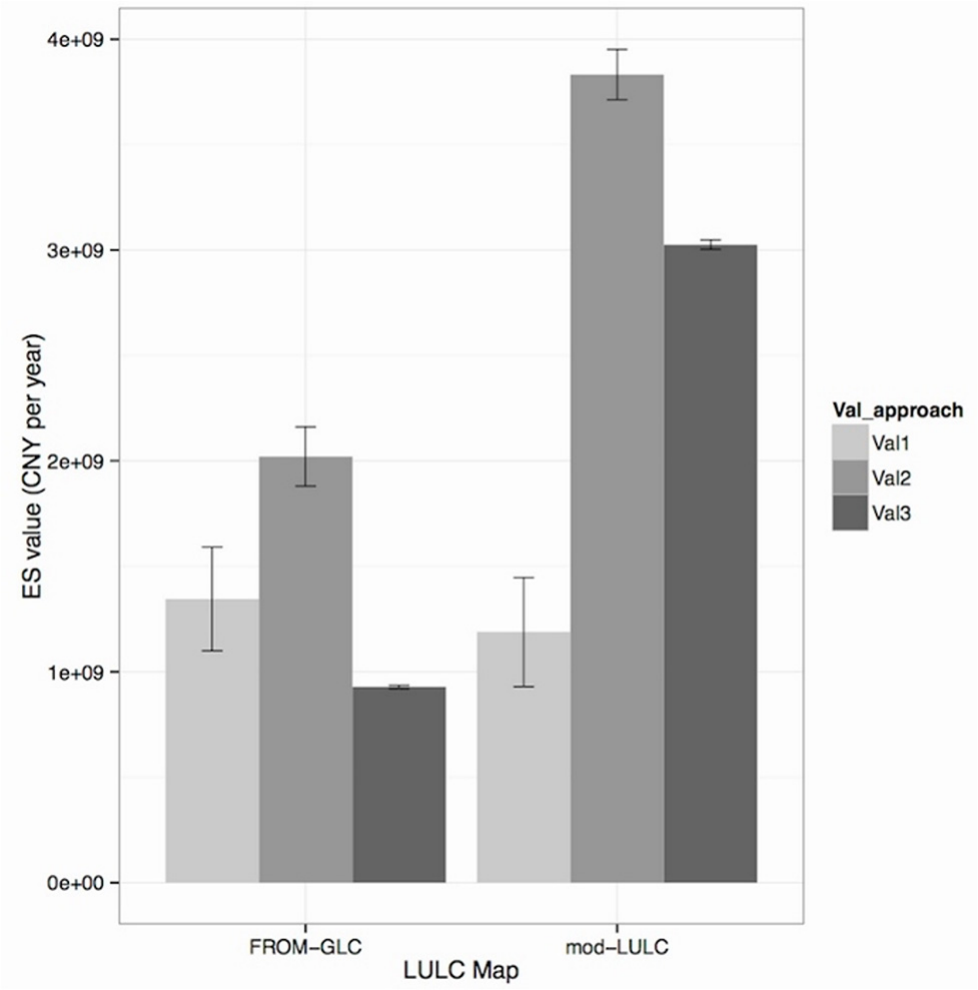
Total ecosystem service value for whole study area using the six different approaches. Val1 = Costanza estimates; Val2 = Amended Costanza estimates; Val3 = Amended estimates only for services identified as locally important; All values given in Chinese Yuan per year; Error bars show variation of some ecosystem services that used low and high estimates provided by Costanza et al. (1997) [1]. Those with small error bars and thus low variation in ESV were calculated using only one estimate; Actual values are provided in S7 Table.

ESV was calculated for the whole study area using the two maps and three valuation approaches (Fig 3). Locally-relevant approaches had less variability than globally derived estimates. For example, using Val3 and the FROM-GLC map we generated an arithmetic range of 14 x 10^6^ CNY yr^-1^. Using Val1 and the FROM-GLC map we generated an arithmetic range of 491 x 10^6^ CNY yr^-1^. This reflects whether services with high and low estimates were included in each approach or not (as provided in the supplementary material of Costanza et al., (1997) [1]; See this paper for a detailed explanation for the variation in their estimates [1]). For both LULC maps, Val2 yielded a greater overall ESV compared to Val1 & Val3, but with greater values for the modified-LULC rather than the FROM-GLC map.

Overall ESV was higher for the modified LULC compared to the FROM-GLC map, even though more forest was classified in the FROM-GLC map–with forest being the most valuable of land classes. However, the total ESV for Forest using Val2 and Val3 approaches, is much greater in the modified-LULC map (2.039 billion CNY yr^-1^ for Forest outside of Core and Buffer zones and using Val2 for example) than that in the FROM-GLC map (3.942 million ± 90,140 CNY yr^-1^ for Needleleaf, 681.8 million ± 35.59 million CNY yr^-1^ for Mixed and 576.1 million CNY yr^-1^ for Broadleaf forest outside of Core and Buffer zones and using Val2 for example). This is because the FROM-GLC map had three sub-classes of Forest: Broadleaf, Mixed and Needleleaf. For the Val2 and Val3 approaches, locally relevant data concerning direct-use services within forests, i.e. bamboo extraction and pine tree plantations, was included. As the modified-LULC map only includes one forest-type, these coefficients for bamboo and pine extraction were applied to every unit area of Forest. For the FROM-GLC map however, pine tree plantation coefficients are only applied to Needleleaf forest, and bamboo extraction only applied to Mixed forest. This therefore reduces the total unit value for the various Forest classes in the FROM-GLC map.

Similarly the total ESV from Farmland for the modified-LULC map was greater than that for the FROM-GLC map, since more land has been classified as Farmland within the modified-LULC (251.96 km^2^) than within the FROM-GLC map (188.18 km^2^). Additionally, in the FROM-GLC map, Farmland is divided into several sub-classes including Bare crop, Rice, Greenhouse crops and Other crops. For the Bare crop and Greenhouse crops, no food production estimates were included as they were assumed to be either “bare” with no crop production, or for the Greenhouse crops, food production estimates were omitted to avoid double-counting of other food production coefficients. These coefficients would otherwise fall into the Rice and Other crops classes. The modified-LULC map applies all agriculturally-related ESV coefficients into every unit area of Farmland, whereas in the FROM-GLC map, coefficients differ for each sub-class. This effect is so great that for the modified-LULC map, the total ESV using the Val3 approach is more than double that using the Val1 approach (Fig 3). This is despite the fact that fewer services have been incorporated into the Val3 compared to the Val1 approach. However, because the Val3 approach included direct-use values calculated from social surveys and applied these coefficients to every unit of Forest or Farmland class in the modified-LULC map, the overall ESV is much greater than if the Val1 approach is used. However, because the modified-LULC map had not sub-divided particular categories (especially for Forest and also for Farmland) and because within the FROM-GLC classification only the Mixed forest included all the direct-use services in the calculation of its coefficient, the latter map showed a much smaller total ESV value in comparison to the modified-LULC map for the Val3 approach. Essentially, this demonstrates a greater impact of different LULC classification approaches compared to different valuation approaches on calculating ESV.

These distinctions are important as these two classes, Forest and Farmland, cover most of the study area (Table 2) and were therefore most responsible for determining total ESV (S6 Table). For example, with the modified-LULC map and using Val2, of the total value, 60.5% was taken up by the ESV of Forest (1.160 billion CNY yr^-1^ on average between Forest inside and outside of the Core or Buffer zones from a total ESV of 3.832 billion CNY yr^-1^), and 36.0% was taken up by the ESV of Farmland (1.376 billion CNY yr^-1^). Other LULC classes made up only small percentages of the total ESV measurements.

### ESV Across Management Zones

**Fig 4:**
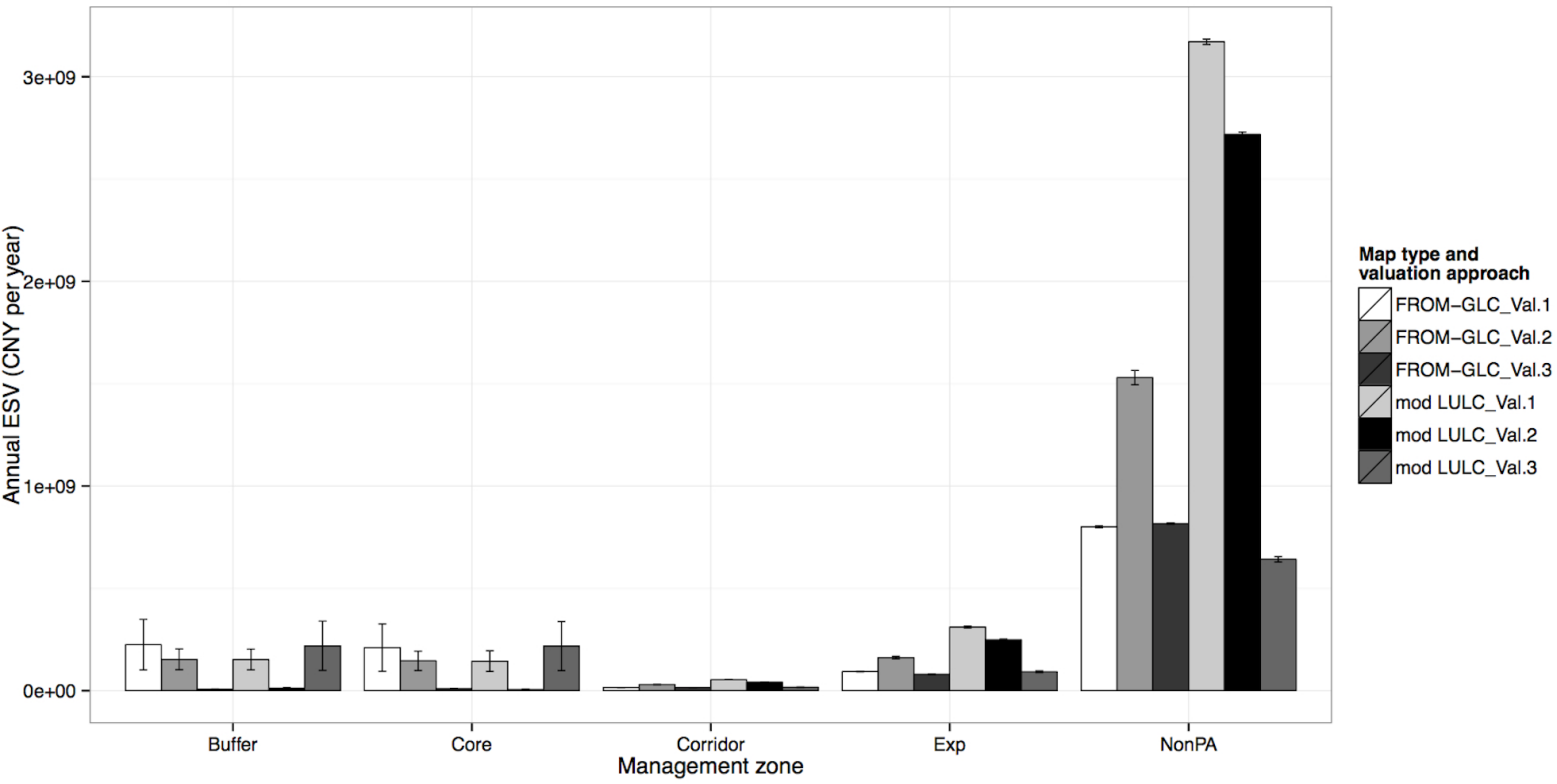
Total ecosystem service value for each management zone for all six valuation approaches. All values given in Chinese Yuan per year; Error bars show variation of some ecosystem services that used low and high estimates provided by Costanza et al. (1997) [1].

In terms of NGH Nature Reserve management, the greatest total ESV is found in the Non-protected area, irrespective of LULC or valuation approaches adopted (Fig 4). The Buffer and Core zones had similar patterns of annual ESV estimates across all approaches (Fig 4), and were dissimilar to other management zones. This is possibly due to the decisions made in constructing different valuation approaches (see S2 Table and S3 Table). These decisions separated these two zones from the others initially by assuming that they would contain Tropical rather than General or Mixed forest types (i.e. different biome definitions from Costanza et al., (1997) [1]) and most importantly that no natural product extraction is allowed in these zones [23]. This chiefly affects Val2 and Val3 approaches, which include locally-derived values based on estimates of raw product extraction. Therefore protected area rules and regulations are shown to make the ESV patterns for Buffer and Core zones and ESV patterns for Corridor, Experimental and Non-protected area zones, distinct.

Adjusting for area, we ranked different zones according to their ESV measurements per unit area (Table 3). The Non-protected area was most valuable for one map and the least valuable for the other. Such patterns are probably due to the LULC typology differences mentioned previously where ESV coefficients were either applied to every unit of Forest or Farmland class, or only particular sub-classes. Also, apart from the approach combining Val2 and the FROM-GLC map, we see that for the more locally-relevant valuation approaches (Val2 and Val3), the zones with least official protection (i.e. Corridor, Experimental and Non-protected area zones) were more valuable compared to the zones with most official protection (i.e. Core and Buffer zones) where extraction of firewood, bamboo and wild foods is prohibited. Conversely, using a globally-relevant valuation approach (Val1), the zones with least official protection were the least valuable and those with most official protection were the most valuable.

**Table 3:** The most and least valuable management zones according to their ecosystem service value per unit area.

	FROM-GLC		Modified-LULC	
	*Most valuable*	*Least valuable*	*Most valuable*	*Least valuable*
**Val. 1 –Costanza**	BUFFER	NonPA	CORE	NonPA
**Val. 2 –Amended Costanza**	CORE	NonPA	NonPA	CORE
**Val. 3 –Locally amended**	NonPA	BUFFER	NonPA	BUFFER

Calculated using each LULC map and valuation approach and based on results shown in Fig 3 and S8 Table; NonPA = Non-protected area.

**Fig 5:**
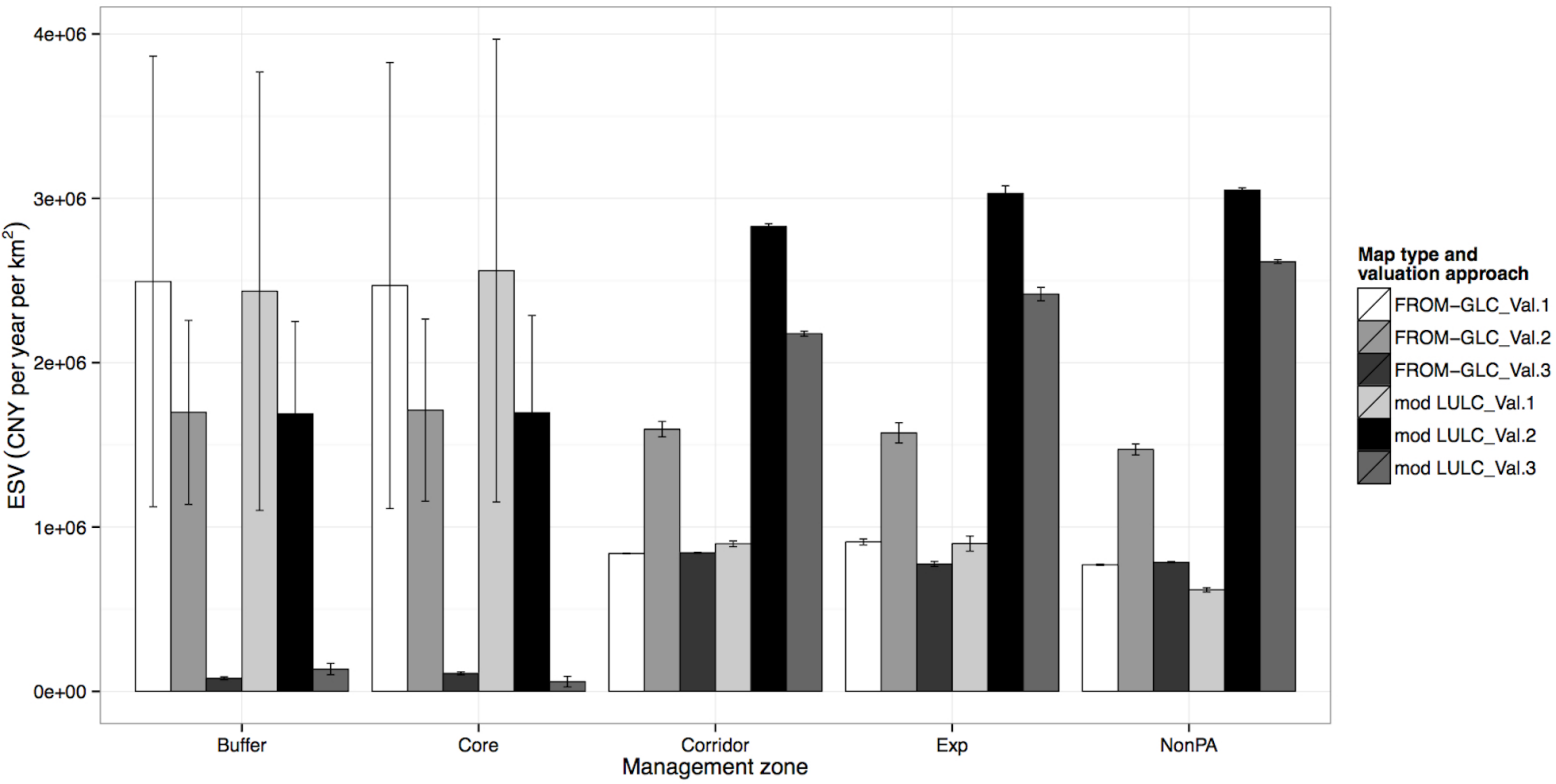
Ecosystem service value per unit area for each management zone and using six valuation approaches. All values given in Chinese Yuan per year per km ^2^; Error bars show variation of some ecosystem services that used low and high estimates provided by Costanza et al. (1997) [1].

The patterns shared by Corridor, Experimental and Non-protected area zones (zones with least official protection) show that the Val2 approach had the highest ESV per unit area for both maps (Fig 5). These values for the modified-LULC map were greater than that for the FROM-GLC map as a result of the subdivision of Forest and Farmland classes in the FROM-GLC map (S6 Table). In each zone and for each map, Forest and Farmland were the predominant LULC classes. Also, unlike the FROM-GLC map, the modified-LULC map contains a separate class for Rubber (Val2 ESV coefficient = 1.638 million CNY yr^-1^ km^-2^), which despite forming only a small proportion of the Corridor, Experimental and Non-protected area zones (15.90%, 3.07% and 2.92% of total area respectively), will also contribute to an increased ESV in the modified-LULC compared to the FROM-GLC map, particularly for locally-relevant valuation approaches (i.e. Val2 and Val3), where income from rubber tapping is included in the ESV estimates. Applying the Val1 approach to both LULC maps and all three of these zones (Corridor, Experimental and Non-protected area) shows much lower ESV estimations compared to other approaches, as the locally-derived direct-use values such as pine plantation, wild food, firewood and bamboo extraction, were not included.

Finally, we observed that using the Val3 approach and the FROM-GLC map, ESV estimation was very close to that for the estimates using the Val1 approach or, in the case of the Experimental zone, slightly less (Fig 5). However, for the modified-LULC map, the Val3 estimation was consistently more than double that of the Val1 estimates for all three zones. This demonstrates that the effect of including raw product extraction values in ESV coefficients (i.e. using the Val3 approach) is not as strong as the effect of using different LULC typologies.

## Conclusion

We have demonstrated that different distribution patterns of ecosystem service value across management zones can be obtained using the six different valuation approaches described. The paper identifies two main aspects of the valuation approaches that have weighted heavily in influencing such patterns across space: the LULC map typology and the valuation approach itself. For example, a large amount of these differences were due to the fact that the FROM-GLC map had divided its Forest class into sub-classes and that the modified-LULC map did not. This was the case whether we explored differences in ESV measurements between management zones, or overall ESV measurements for the whole study area. Similarly, ESV estimates using the modified-LULC map may have changed if LULC class resolution were improved, and was more relevant to the local situation (e.g. divide the Farmland class into Tea, Banana and Rice). The FROM-GLC map, taken from a global dataset, was not built with local conditions of the NGH Nature Reserve in mind. When the modified-LULC map was built, however, it was not possible to distinguish between different crop or forest types using the data and techniques available to Nature Reserve management staff. Essentially, it is difficult to conclude which map was most accurate. For example, the FROM-GLC map contained three types of forest in its classification [28], but it did not include rubber, which is a very locally important resource. Similarly, the FROM-GLC map contained sub-classes of farmland. However, some of those sub-classes were not necessarily appropriate for this specific area.

As Troy and Wilson (2006) [6] also argue, the building of the LULC map becomes just as important as the methods used for calculating the ecosystem service value estimates themselves. Indeed, Konarska et al., (2002) [36] have highlighted the importance of spatial scale of the LULC base maps used in valuation assessments. One can therefore identify what might make our LULC maps most accurate and locally appropriate for ESV assessments. However, when creating such detailed maps is not possible under particular practical limitations, it is still important to understand how results might vary using other more accessible methods and datasets.

As mentioned previously, it was important to design these six approaches to incorporate both locally and globally-derived data sources and methods that varied according to time, access and skill limitations: Creating the FROM-GLC map required very little data manipulation, was fast and easy to prepare and was built on a globally-derived dataset. The modified LULC map in contrast required labour and time-intensive preparation and was built on locally-relevant criteria. The important distinguishing factor in this case is scale: It may be easier and more accessible to work with methods and data already derived in previous studies. However, acknowledging local idiosyncrasies and the fact that ecosystem service evaluations in particular, are scale-dependent, is important [2,15]. With this in mind, incorporating local knowledge of land-use practices and legislation was a useful way to try and make some of the six approaches most relevant to the local situation. This was done for example, in assessing the inclusion/exclusion of some services when calculating ESV for different management zones with different activities occurring in each.

It was not the purpose of this paper to investigate the most accurate or appropriate valuation techniques, as this has been done elsewhere (For example [15,37–40]). Indeed other work (e.g. [41,42]) has also explained the limitations and inaccuracies of applying the core method used by Costanza et al., (1997) [1] in, more specifically, the inappropriate use of the benefit transfer approach. For this reason we urge readers to treat the actual values calculated in this paper, with caution. Rather, it was more important in this paper to demonstrate the different results that could be obtained using valuation approaches that were available to reserve managers and their support teams in remote China and other developing regions. It appears possible that the LULC class typology has a more influential role on ESV estimates than the valuation approach. However, the effect of different valuation approaches is still present (e.g. Fig 4), and must still be acknowledged in ESV assessments.

We also explored which approaches should be used under certain conditions, and for this particular locality. This greatly depends on the purpose of the assessment itself and the resources available to those conducting the assessment [43]. For example, if a human development initiative where improving access to benefits for local people were the focus, then the Val3 approach might be considered most appropriate, as it takes local preferences into consideration. As has been shown above, this would identify areas outside of the protected area and the Experimental zone as more valuable in terms of ESV for either LULC map used.

We have also mentioned that the Corridor zone is not presently under specific management, but has been highlighted in biodiversity conservation plans, as a potential area for future habitat restoration. If an ESV assessment was to show that this area is less valuable than the Non-protected areas when considering just local benefits, then it could mean that future management would have less of an overall negative impact on local people if it were to be transformed into a protected functioning ecological corridor. This is indeed the case for Val2 & 3 approaches, but only for the modified-LULC map. If management focused on global benefits however, then our results would suggest that highly protected areas (i.e. Core and Buffer zones) would be most valuable per unit area. Selecting the appropriate ESV assessments according to the stated purpose and reason for undertaking, could be crucial for protected area management decisions for NGH Nature Reserve and its surrounding area [2].

A manager for this local area may also want to know which zones are most valuable, and in this case might immediately look towards the Non-protected areas (Fig 4). However, we have demonstrated the effect of area size, and so observing ESV measurements per unit area yields quite different results (Fig 5). This might be important if a manager were to incorporate cost-benefit analyses (e.g. [44]) where a greater value of ESV can be protected for the least management costs (if such costs are dependent on area size). Observing how ESV measurements per unit area vary between management zones has helped explain why such differences are present. This was clearly demonstrated by the difference between two groups of zones: those with greatest official protection and those with least official protection. As mentioned, this was due to the way by which forest was defined as being more valuable in the protected compared to the least protected zones. Also, in the highly protected zones, based on local regulations we know that extraction of raw products is not allowed and so any value obtained from that would not be included in their value assessments. This not only reflects the importance of definitions and methodological decisions in valuation approaches. It also demonstrates the application of local knowledge on the valuation assessment process, to make it as relevant as possible to local conditions.

Considering the effect of different LULC maps and their associated typologies and different valuation approaches on ESV estimates, it is clear that drawing solid, confident conclusions for reserves, in terms of which management zones hold the greatest ecosystem service value, is prone to difficulties. Furthermore in developing regions, land-use change is occurring rapidly [45]. For example in NGH, roads are continually being improved, providing increased access for business, agriculture and tourism, and plantations continue to expand their coverage. Such changes would be expected to impact ESV estimates [18], and so any ESV results will be time-bound to the point at which they were calculated.

Our paper provides a useful insight into the results that can be yielded and the management-based interpretations that can be drawn, using methods and data available to nature reserve managers. We demonstrate that different methods for valuing ecosystem services do yield different valuation results. Such methods might be based on locally or globally-derived data or resource-intensive or straight-forward methods of data access. This could have serious implications on management decisions for protected areas when, for example, managers wish to act on zones with most or least value, and calculating such value can differ so widely depending on the methods used. It is difficult to precisely explain the direction of this variation when we have demonstrated an inconsistent effect of using either different LULC base map or using different valuation approaches. Understanding the nature of such effects in greater detail would certainly be worthwhile. However, we are able to conclude from our study that this variation appears to be largely associated with choice of LULC class typology as well as the decisions used to shape the adopted economic valuation approach. Until standardized protocols for ecosystem service valuation have been agreed upon and are readily available for use at variable scales, managers and decision-makers should be aware of the caveats associated with using different approaches.

## Supporting Information

S1 File Raw data used to calculate value of crops, timber, firewood, bamboo and all other non-forest products. (XLSX)

S1 Table Values used to calculate food production service values from social survey data. All amounts are given to 2 d.p.^a b^
(DOC)

S2 Table Detailed rules for three valuation approaches for FROM-GLC LULC classes. Any text in italics highlights data derived from 2012 and 2013 social surveys, otherwise the estimates are from Costanza et al., (1997) [1].^a^
(DOC)

S3 Table Detailed rules for three valuation approaches for modified-LULC classes. Any text in italics highlights data derived from 2012 and 2013 social surveys, otherwise the estimates are from Costanza et al., (1997) [1].
(DOC)

S4 Table Areas of different LULC classes for FROM-GLC map. Recorded in km^2^ (percentages in brackets) and rounded to 3 d.p.
(DOC)

S5 Table Areas of different LULC classes for modified-LULC map. Recorded in km^2^ (percentages in brackets) and rounded to 3 d.p.
(DOC)

S6 Table ESV Coefficients for each class for each LULC map and for all three valuation approaches (including low and high estimates following style of Costanza et al., (1997) [1]); All units in CNY per year per km^2^. (DOC)

S7 Table Total ecosystem service value for whole study area using six different ESV scenarios. (All values in CNY per year).
(DOC)

S8 Table Ecosystem service value per unit area for each management zone using six different ESV scenarios. (All values in CNY per year per km^2^).
(DOC)
