# Supplementary material for: Ecosystem Service Valuation Assessments for Protected Area Management: A Case Study Comparing Methods Using Different Land Cover Classification and Valuation Approaches
Source: PLoS One. 2015 Jun 18;10(6):e0129748. doi: 10.1371/journal.pone.0129748 (PMC4472837; doi:10.1371/journal.pone.0129748)
Supplement: S1 Table — All amounts are given to 2 d.p.a b (DOC) [file pone.0129748.s002.doc]

**S1 Table. Values used to calculate food production service values from social survey data.** All amounts are given to 2 d.p.^a b^

| **Service** | **No. of households contributing to calculation of average rates** | **Coefficient estimate (CNY per km^2^ per year)** |
| --- | --- | --- |
| Rice production | 85 | 1,714,020.00 |
| Tea production | 134 | 860,921.35 |
| Bamboo harvest | NA^c^ | 1,746,000.00 |
| Non-rice crop production (including cassava, corn, hangu (local dry crop), sugarcane, vegetables) | Cassava (66), Corn (63), Hangu (10), Sugarcane (15), Vegetables (18) | 2,850,000.00 |
| Firewood | 6 | 691.29 |
| Wild food (including wild vegetables and bamboo shoots) | 55 | 55.82 |
| Rubber | 23 | 866,646.99 |
| Pine forest | 22 | 690,000.00 |

^a^ It should be noted that estimates were produced that represent a unit area and so it assumes that every unit area of that specific LULC class can be cultivated. Although some of the harvest might be consumed by the household, we have still used the market sale price to value everything cultivated. Also, rice and vegetables are very rarely sold in the area, so in these cases, we used the average purchase price to estimate their value.

^b^ For firewood and wild food, estimates were calculated based on consumption rather than production. To extrapolate these household survey-obtained estimates to all villages, we assumed that each village has an active radius of 5km from the village based on expert local knowledge. We could therefore calculate the average consumption of that village per km^2^ so that our estimates could easily be converted to coefficients per unit area of land.

^c^Market value of individual bamboo trees and estimated number of trees per km^2^ provided by Nature Reserve authority.
