# Supplementary material for: Ecosystem Service Valuation Assessments for Protected Area Management: A Case Study Comparing Methods Using Different Land Cover Classification and Valuation Approaches
Source: PLoS One. 2015 Jun 18;10(6):e0129748. doi: 10.1371/journal.pone.0129748 (PMC4472837; doi:10.1371/journal.pone.0129748)
Supplement: S2 Table — Any text in italics highlights data derived from 2012 and 2013 social surveys, otherwise the estimates are from Costanza et al., (1997) [1].a (DOC) [file pone.0129748.s003.doc]

**S2 Table. Detailed rules for three valuation approaches for FROM-GLC LULC classes**. Any text in italics highlights data derived from 2012 and 2013 social surveys, otherwise the estimates are from Costanza et al., (1997) [1]^a^.

| **Land cover categories (as provided by Gong et al., (2013)** [28]**)** | **Rules** | **Val. 1. Costanza estimate** | **Val. 2. Amended Costanza estimate** | **Val. 3. Locally relevant amended estimate** |
| --- | --- | --- | --- | --- |
| **Bare crop** | Assume there is no crop production | Use ‘cropland’ | Use ‘pollination’ + ‘biological control’ | 0 |
| **Broadleaf (Inside Core + Buffer management zones)** | Distinguishing between management zones is to divide by forest type categories for Costanza et al., (1997) [1] estimates + and for Nature Reserve rules of extraction, where no extraction can take place in Core and Buffer zones; Firewood and wild food extraction values have been calculated for appropriate management zones for the whole study area – see below | Use ‘tropical forest’ | Use ‘tropical forest’ – ‘recreation’ – ‘food production’ – ‘raw products’ | ‘Water supply’ + ‘water regulation’ + ‘culture’ + ‘gas regulation’ |
| **Broadleaf (Outside Core + Buffer management zones)** |  | Use ‘general forest’ | ‘General forest’ – ‘recreation’ + *firewood and wild food* | ‘Water supply’ + ‘water regulation’ + ‘culture’ + ‘gas regulation’ + *firewood + wild food* |
| **Cloud** | Assume no ES value | 0 | 0 | 0 |
| **Greenhouse crops** | For Val. 2 and Val. 3, no food production estimates are included as they are accounted for in other LULC classes, and there are also no GH crops known in this area. | Use ‘cropland’ | ‘Pollination’ + ‘biological control’ | 0 |
| **Grassland** | During social surveys there was no reported food production or extraction of any product from grasslands. | Use ‘grassland’ | Use ‘Grassland’– ‘recreation’ – ‘food production’ | ‘Water supply’ + ‘water regulation’ + ‘culture’ + ‘gas regulation’ |
| **Gravel** | Assume no ES value | 0 | 0 | 0 |
| **High/Low Albedo** | Assume no ES value | 0 | 0 | 0 |
| **Lake** | Food production not included for Val. 3: not identified during social survey | Use ‘Lakes/Rivers’ | Use ‘Lakes/Rivers’ | ‘Water regulation’ + ‘water supply’ + ‘cultural’ |
| **Mixed Forest (Inside Core + Buffer management zones)** | As above for “Broadleaf Forest” rules | Use ‘tropical’ | Use ‘tropical’ – ‘recreation’ – ‘food production’ – ‘raw products’ | ‘Water supply’ + ‘water regulation’ + ‘culture’ + ‘gas regulation’ |
| **Mixed Forest (Outside Core + Buffer management zones)** |  | Use ‘general forest’ | Use ‘General forest’ – ‘recreation’ – ‘food production’ – ‘raw products’+ *firewood + bamboo + wild food* | ‘Water supply’ + ‘water regulation’ + ‘culture’ + ‘gas regulation’ *+ firewood + wild food + bamboo* |
| **Needleleaf (Inside Core + Buffer management zones)** | As above for “Broadleaf Forest” rules | Use ‘temperate forest’ | Use ‘temperate’ – ‘recreation’ – ‘food production’ – ‘raw products’ | ‘Water supply’ + ‘water regulation’ + ‘culture’ + ‘gas regulation’ |
| **Needleleaf (Outside Core + Buffer management zones)** |  | Use ‘temperate forest’ | Use ‘Temperate forest’ – ‘recreation’ – ‘food production’ – ‘raw products’ *+ firewood + pine plantation + wild food* | ‘Water supply’ + ‘water regulation’ + ‘culture’ + ‘gas regulation’ *+ firewood + pine plantation + wild food* |
| **Orchard** | Assume these are tea and rubber plantations. | Use ‘Cropland’ (as did Hu et al., (2008) [32] | Use ‘cropland’ – ‘food production’ *+ tea + rubber* | *Tea + rubber* |
| **Other bare land** | Assume no ES value | 0 | 0 | 0 |
| **Other crop** |  | Use ‘cropland’ | ‘Pollination’ + ‘biological control’ + *non-rice crop production* | *Non-rice crop production* |
| **Pond** | Treating these the same as Lakes and rivers but assuming no food production or recreation. | Use ‘Lakes/Rivers’ | Use ‘Lakes/Rivers’ – ‘food production’ - ‘recreation’ | ‘Water supply’ + ‘water regulation’ + ‘culture’ |
| **Rice** |  | Use ‘cropland’ | ‘Pollination’ + ‘biological control’ + *rice production* | *Rice production* |
| **River** | Food production not included for Val. 3: not identified during social survey | Use ‘Lakes/Rivers’ | Use ‘Lakes/Rivers’ | ‘Water supply’ + ‘water regulation’ + ‘culture’ |
| **Shrub** |  | Use ‘Grassland’ | Use ‘Grassland’ – ‘recreation’ – ‘food production’ | ‘Water supply’ + ‘water regulation’ + ‘culture’ + ‘gas regulation’ |

^a^The value coefficients taken from Costanza et al., (1997) [1] also had to be standardized to 2013 CNY equivalents per km^2^. This was done firstly by calculating for inflation using information from the US Bureau of Labor Statistics (1 US $ in 1994 is equivalent to 1.57 US $ in 2013); Secondly, to ensure the same currency was used and to match the estimations taken from the social surveys, an exchange rate of 0.1603111985 US $ to 1 CNY was used (correct on 1st Jan 2013 by XE Currency Converter).
