# Supplementary material for: Ecosystem Service Valuation Assessments for Protected Area Management: A Case Study Comparing Methods Using Different Land Cover Classification and Valuation Approaches
Source: PLoS One. 2015 Jun 18;10(6):e0129748. doi: 10.1371/journal.pone.0129748 (PMC4472837; doi:10.1371/journal.pone.0129748)
Supplement: S3 Table — Any text in italics highlights data derived from 2012 and 2013 social surveys, otherwise the estimates are from Costanza et al., (1997) [1]. (DOC) [file pone.0129748.s004.doc]

**S3 Table. Detailed rules for three valuation approaches for modified-LULC classes**. Any text in italics highlights data derived from 2012 and 2013 social surveys, otherwise the estimates are from Costanza et al., (1997) [1]

| **Land cover categories** | **Rules** | **Val. 1. Costanza estimate** | **Val. 2. Amended Costanza estimate** | **Val. 3. Locally relevant amended estimate** |
| --- | --- | --- | --- | --- |
| **Buildings** | Assume no ES value | 0 | 0 | 0 |
| **Road** | Assume no ES value | 0 | 0 | 0 |
| **Forest (Inside Core + Buffer management zones)** | Not including rubber forest | Use ‘tropical’ | Use ‘tropical’ – ‘recreation’ – ‘food prod’ – ‘raw’ | ‘Water supply’ + ‘water regulation’ + ‘culture’ + ‘gas regulation’ |
| **Forest (Outside Core + Buffer management zones)** | Not including rubber forest | Use ‘general forest’ | ‘General forest’ – ‘recreation’ – ‘food prod’ – ‘raw’ + *firewood + bamboo + wild food + pine plantation* | ‘Water supply’ + ‘water regulation’ + ‘culture’ + ‘gas regulation’ *+ firewood + wild food + bamboo + pine plantation* |
| **Rubber** |  | Use ‘general forest’ | Use ‘General forest’ – ‘food prod’ – ‘raw’ *+ rubber production*  (similar to Hu et al., (2008) [37] | ‘Water supply’ + ‘water regulation’ + ‘culture’ + ‘gas regulation’ *+ rubber production* |
| **Bare land** | Assume no ES value | 0 | 0 | 0 |
| **Cropland** | Include rice and tea together | Use ‘cropland’ | ‘Pollination’ + ‘bio control’ + *rice + tea + non-rice crop production* | *Rice + tea + non-rice crop production* |
| **Water bodies** | Food production not included for Val. 3: not identified during social survey | Use ‘Lakes/Rivers’ | Use ‘Lakes/Rivers’ | ‘Water regulation’ + ‘water supply’ + ‘cultural’ |
| **River** | Food production not included for Val. 3: not identified during social survey | Use ‘Lakes/Rivers’ | Use ‘Lakes/Rivers’ | ‘Water regulation’ + ‘water supply’ + ‘cultural’ |
| **Grassland** |  | Use ‘grassland’ | Use ‘Grassland’ – ‘recreation’ – ‘food production’ | ‘Water supply’ + ‘water regulation’ + ‘culture’ + ‘gas regulation’ |
| **Shrub** |  | Use ‘grassland’ | Use ‘Grassland’ – ‘recreation’ – ‘food production’ | ‘Water supply’ + ‘water regulation’ + ‘culture’ + ‘gas regulation’ |
