# Supplementary material for: Ecosystem Service Valuation Assessments for Protected Area Management: A Case Study Comparing Methods Using Different Land Cover Classification and Valuation Approaches
Source: PLoS One. 2015 Jun 18;10(6):e0129748. doi: 10.1371/journal.pone.0129748 (PMC4472837; doi:10.1371/journal.pone.0129748)
Supplement: S4 Table — Recorded in km2 (percentages in brackets) and rounded to 3 d.p. (DOC) [file pone.0129748.s005.doc]

**S4 Table. Areas of different LULC classes for FROM-GLC map.** Recorded in km^2^ (percentages in brackets) and rounded to 3 d.p.

| **Land category** | **Core** | **Buffer** | **Experimental** | **NonPA** | **Corridor** | **Total** |
| --- | --- | --- | --- | --- | --- | --- |
| **Bare crop** | 0.018  (0.021) | 0.018  (0.019) | 0.273  (0.266) | 31.067  (2.989) | 0.152  (0.804) | 31.527 |
| **Broadleaf forest inside Core & Buffer** | 49.399  (57.970) | 52.307  (58.009) | - | - | - | 101.706 |
| **Broadleaf forest outside Core & Buffer** | - | - | 60.653  (59.060) | 580.267  (55.836) | 9.990  (52.927) | 650.909 |
| **Cloud** | - | - | 0.007  (0.007) | 0.213  (0.020 | 0.001  (0.005) | 0.221 |
| **Greenhouse crops** | 0.003  (0.003) | 0.002  (0.002) | 0.004  (0.004) | 0.056  (0.005) | 0.005  (0.027) | 0.069 |
| **Grassland** | 0.071  (0.083) | 0.145  (0.161) | 0.968  (0.943) | 54.787  (5.272) | 0.747  (3.956) | 56.719 |
| **Gravel** | 0.001  (0.001) | - | - | 0.013  (0.001) | - | 0.013 |
| **High albedo** | - | - | - | 0.064  (0.006) | - | 0.064 |
| **Low albedo** | 0.001  (0.001) | - | 0.001  (0.001) | 0.201  (0.019) | - | 0.202 |
| **Lake** | - | - | 0.369  (0.359) | 0.113  (0.011) | - | 0.482 |
| **Mixed forest inside Core & Buffer** | 32.661  (38.327) | 35.445  (39.309) | - | - | - | 68.106 |
| **Mixed forest outside Core & Buffer** | - | - | 33.231  (32.358) | 223.872  (21.542) | 6.261  (33.174) | 263.364 |
| **Needleleaf forest inside Core & Buffer** | 0.281  (0.330) | 0.268  (0.297) | - | - | - | 0.549 |
| **Needleleaf forest outside Core & Buffer** | - | - | 1.089  (1.060) | 3.371  (0.324) | 0.030  (0.159) | 4.490 |
| **Orchard** | - | 0.001  (0.001) | 0.001  (0.001) | 0.012  (0.001) | 0.000  (0.000) | 0.014 |
| **Other bare land** | - | - | - | 0.058  (0.006) | - | 0.058 |
| **Other crop** | 2.767  (3.247) | 1.978  (2.194) | 6.066  (5.907) | 143.142  (13.774) | 1.659  (8.787) | 155.613 |
| **Pond** | - | - | 0.002  (0.002) | 0.004  (0.000) | - | 0.006 |
| **Rice** | 0.008  (0.010) | 0.005  (0.005) | 0.018  (0.018) | 0.921  (0.089) | 0.022  (0.119) | 0.974 |
| **River** | 0.005  (0.006) | 0.000  (0.000) | 0.009  (0.008) | 0.863  (0.083) | 0.008  (0.042) | 0.885 |
| **Shrub** | - | 0.001  (0.001) | 0.006  (0.006) | 0.202  (0.019) | - | 0.210 |
| **TOTAL** | 85.215 | 90.170 | 102.696 | 1,039.227 | 18.874 |  |
