# Supplementary material for: Ecosystem Service Valuation Assessments for Protected Area Management: A Case Study Comparing Methods Using Different Land Cover Classification and Valuation Approaches
Source: PLoS One. 2015 Jun 18;10(6):e0129748. doi: 10.1371/journal.pone.0129748 (PMC4472837; doi:10.1371/journal.pone.0129748)
Supplement: S5 Table — Recorded in km2 (percentages in brackets) and rounded to 3 d.p. (DOC) [file pone.0129748.s006.doc]

**S5 Table. Areas of different LULC classes for modified-LULC map. Recorded in km^2^ (percentages in brackets) and rounded to 3 d.p.**

| **Land category** | **Core** | **Buffer** | **Experimental** | **NonPA** | **Corridor** | **Total** |
| --- | --- | --- | --- | --- | --- | --- |
| **Road and building** | - | 0.007  (0.008) | 0.171  (0.166) | 15.078  (1.451) | 0.015  (0.078) | 15.271 |
| **Road** | - | 0.031  (0.034) | 0.176  (0.171) | 0.963  (0.093) | - | 1.170 |
| **Forest inside Core & Buffer** | 83.582  (98.085) | 83.474  (92.588) | - | - | - | 167.056 |
| **Forest outside Core & Buffer** | - | - | 82.613  (80.450) | 552.150  (53.127) | 13.886  (73.657) | 648.648 |
| **Rubber** | 0.017  (0.020) | 0.029  (0.032) | 3.148  (3.065) | 30.340  (2.919) | 2.997  (15.898) | 36.531 |
| **Bare land** | 0.002  (0.003) | 0.159  (0.176) | 0.044  (0.043) | 1.322  (0.127) | - | 1.527 |
| **Farmland** | 0.010  (0.012) | 1.217  (1.349) | 6.663  (6.489) | 243.339  (23.414) | 0.729  (3.867) | 251.957 |
| **Water** | - | 0.005  (0.005) | 0.310  (0.302) | 0.622  (0.060) | - | 0.937 |
| **River** | 0.478  (0.561) | 0.554  (0.614) | 0.691  (0.673) | 2.163  (0.208) | 0.069  (0.368) | 3.955 |
| **Grassland** | - | 0.043  (0.047) | 0.320  (0.312) | 1.124  (0.108) | - | 1.487 |
| **Scrub** | 1.125  (1.320) | 4.638  (5.144) | 8.552  (8.328) | 192.207  (18.494) | 1.156  (6.131) | 207.678 |
| **TOTAL** | 85.214 | 90.156 | 102.689 | 1,039.307 | 18.852 |  |
