# Supplementary material for: Ecosystem Service Valuation Assessments for Protected Area Management: A Case Study Comparing Methods Using Different Land Cover Classification and Valuation Approaches
Source: PLoS One. 2015 Jun 18;10(6):e0129748. doi: 10.1371/journal.pone.0129748 (PMC4472837; doi:10.1371/journal.pone.0129748)
Supplement: S6 Table — (DOC) [file pone.0129748.s007.doc]

**S6 Table. ESV Coefficients for each class for each LULC map and for all three valuation approaches** (including low and high estimates following style of Costanza et al., (1997) [1]); All units in CNY per year per km^2^.

| **Land category** | **Val. 1 Low** | **Val. 1 High** | **Val. 2 Low** | **Val. 2 High** | **Val. 3 Low** | **Val. 3 High** |
| --- | --- | --- | --- | --- | --- | --- |
| **FROM-GLC-Bare crop** | 90,099.76 | 90,099.76 | 37,215.12 | 37,215.12 | - | - |
| **FROM-GLC-Broadleaf forest inside Core & Buffer** | 1,150,828.52 | 3,968,404.62 | 1,102,938.54 | 2,253,767.07 | 8,030.63 | 26,442.32 |
| **FROM-GLC-Broadleaf forest outside Core & Buffer** | 948,985.48 | 948,985.48 | 885,095.81 | 885,095.81 | 7,602.53 | 7,602.53 |
| **FROM-GLC-Cloud** | - | - | - | - | - | - |
| **FROM-GLC-Greenhouse crops** | 90,099.76 | 90,099.76 | 37,215.12 | 37,215.12 | - | - |
| **FROM-GLC-Grassland** | 228,099.29 | 228,099.29 | 170,278.75 | 170,278.75 | 9,342.95 | 9,342.95 |
| **FROM-GLC-Gravel** | - | - | - | - | - | - |
| **FROM-GLC-High albedo** | - | - | - | - | - | - |
| **FROM-GLC-Low albedo** | - | - | - | - | - | - |
| **FROM-GLC-Lake** | 3,580,485.99 | 13,064,464.74 | 3,580,485.99 | 13,064,464.74 | 3,222,045.65 | 11,589,570.89 |
| **FROM-GLC-Mixed forest inside Core & Buffer** | 1,150,828.52 | 3,968,404.62 | 1,102,938.54 | 2,253,767.07 | 8,030.63 | 26,442.32 |
| **FROM-GLC-Mixed forest outside Core & Buffer** | 948,985.48 | 948,985.48 | 2,453,834.33 | 2,724,133.60 | 1,753,602.53 | 1,753,602.53 |
| **FROM-GLC-Needleleaf forest inside Core & Buffer** | 254,815.82 | 336,591.15 | 167,272.16 | 207,425.31 | 97.93 | 4,015.32 |
| **FROM-GLC-Needleleaf forest outside Core & Buffer** | 254,815.82 | 336,591.15 | 858,019.27 | 898,172.42 | 690,845.04 | 694,762.43 |
| **FROM-GLC-Orchard** | 90,099.76 | 90,099.76 | 1,764,783.46 | 1,764,783.46 | 1,727,568.34 | 1,727,568.34 |
| **FROM-GLC-Other bare land** | - | - | - | - | - | - |
| **FROM-GLC-Other crop** | 90,099.76 | 90,099.76 | 2,887,215.12 | 2,887,215.12 | 2,850,000.00 | 2,850,000.00 |
| **FROM-GLC-Pond** | 3,580,485.99 | 13,064,464.74 | 3,315,083.44 | 12,799,062.19 | 3,222,045.65 | 11,589,570.89 |
| **FROM-GLC-Rice** | 90,099.76 | 90,099.76 | 1,751,235.12 | 1,751,235.12 | 1,714,020.00 | 1,714,020.00 |
| **FROM-GLC-River** | 3,580,485.99 | 13,064,464.74 | 3,580,485.99 | 13,064,464.74 | 3,222,045.65 | 11,589,570.89 |
| **FROM-GLC-Shrub** | 228,207.01 | 228,207.01 | 170,386.47 | 170,386.47 | 9,342.95 | 9,342.95 |
| **Modi-LULC-Road and building** | - | - | - | - | - | - |
| **Modi-LULC-Road** | - | - | - | - | - | - |
| **Modi-LULC-Forest inside Core & Buffer** | 1,150,828.52 | 3,968,404.62 | 1,102,938.54 | 2,253,767.07 | 8,030.63 | 26,442.32 |
| **Modi-LULC-Forest outside Core & Buffer** | 948,985.48 | 948,985.48 | 3,143,834.33 | 3,143,834.33 | 2,443,602.53 | 2,443,602.53 |
| **Modi-LULC-Rubber** | 948,985.48 | 948,985.48 | 1,638,370.99 | 1,638,370.99 | 873,502.41 | 873,502.41 |
| **Modi-LULC-Bare land** | - | - | - | - | - | - |
| **Modi-LULC-Farmland** | 90,099.76 | 90,099.76 | 5,462,156.47 | 5,462,156.47 | 5,424,941.35 | 5,424,941.35 |
| **Modi-LULC-Water** | 3,580,485.99 | 13,064,464.74 | 3,580,485.99 | 13,064,464.74 | 3,222,045.65 | 11,589,570.89 |
| **Modi-LULC-River** | 3,580,485.99 | 13,064,464.74 | 3,580,485.99 | 13,064,464.74 | 3,222,045.65 | 11,589,570.89 |
| **Modi-LULC-Grassland** | 228,099.29 | 228,099.29 | 170,278.75 | 170,278.75 | 9,342.95 | 9,342.95 |
| **Modi-LULC-Scrub** | 228,099.29 | 228,099.29 | 170,278.75 | 170,278.75 | 9,342.95 | 9,342.95 |
