# Supplementary material for: Ecosystem Service Valuation Assessments for Protected Area Management: A Case Study Comparing Methods Using Different Land Cover Classification and Valuation Approaches
Source: PLoS One. 2015 Jun 18;10(6):e0129748. doi: 10.1371/journal.pone.0129748 (PMC4472837; doi:10.1371/journal.pone.0129748)
Supplement: S7 Table — (All values in CNY per year). (DOC) [file pone.0129748.s008.doc]

**S7 Table. Total ecosystem service value for whole study area using six different ESV scenarios.** (All values in CNY per year)

| **MAP** | **Val1 - Costanza** | **Val2 – Amended Cost** | **Val3 - Local** |
| --- | --- | --- | --- |
| **FROM-GLC** | 1,345,152,176.80  ±  245,950,485.99 | 2,020,332,680.52  ±  139,922,373.21 | 928,720,397.31  ±  7,321,554.03 |
| **Modified-LULC** | 1,188,950,518.55  ±  258,544,575.90 | 3,832,034,456.03  ±  119,324,348.06 | 3,024,865,811.54  ±  22,004,891.97 |
