# Supplementary material for: Ecosystem Service Valuation Assessments for Protected Area Management: A Case Study Comparing Methods Using Different Land Cover Classification and Valuation Approaches
Source: PLoS One. 2015 Jun 18;10(6):e0129748. doi: 10.1371/journal.pone.0129748 (PMC4472837; doi:10.1371/journal.pone.0129748)
Supplement: S8 Table — (All values in CNY per year per km2). (DOC) [file pone.0129748.s009.doc]

**S8 Table. Ecosystem service value per unit area for each management zone using six different ESV scenarios.** (All values in CNY per year per km^2^)

| Map & valuation approach | Core | Buffer | Experimental | Non-protected Area | Corridor |
| --- | --- | --- | --- | --- | --- |
| FROM-GLC Val. 1 | 2469493.81 ± 1357062.14 | 2494258.06 ± 1371148.42 | 909182 ± 17955.07 | 770384.76 ± 4608.25 | 838878.36 ± 2075.82 |
| FROM-GLC  Val. 2 | 1711430.91 ± 554475.89 | 1697673.5 ± 560058.84 | 1573138.95 ± 61466.69 | 1472010.54 ± 33654.92 | 1595072.61 ± 46876.73 |
| FROM-GLC  Val. 3 | 109794.28 ± 9136.57 | 79457.22 ± 8977.23 | 775393.91 ± 15479.65 | 785835.77 ± 3955.11 | 842841.93 ± 1777.17 |
| Modified-LULC  Val. 1 | 2560503 ± 1408414.85 | 2434843.42 ± 1333760.21 | 899275.57 ± 46250.32 | 617691.45 ± 12704 | 897965.93 ± 17453.17 |
| Modified-LULC Val. 2 | 1696106.31 ± 590998.13 | 1688603.78 ± 562152.46 | 3029743.63 ± 46250.32 | 3050902.22 ± 12704 | 2828425.12 ± 17453.17 |
| Modified-LULC Val. 3 | 59373.72 ± 32499.66 | 135819.7 ± 34449.69 | 2417700.33 ± 40805.73 | 2615456.76 ± 11208.48 | 2176375.3 ± 15398.58 |
